# Characterizing Bone Phenotypes Related to Skeletal Fragility Using Advanced Medical Imaging

**DOI:** 10.1007/s11914-023-00830-6

**Published:** 2023-10-26

**Authors:** Danielle E. Whittier, Melissa S. A. M. Bevers, Piet P. M. M. Geusens, Joop P. van den Bergh, Leigh Gabel

**Affiliations:** 1 McCaig Institute for Bone and Joint Health and Alberta Children’s Hospital Research Institute, University of Calgary, Calgary, Canada; 2 Department of Cell Biology and Anatomy, University of Calgary, Calgary, Canada; 3 Department of Internal Medicine, VieCuri Medical Center, Venlo, The Netherlands; 4 NUTRIM School for Nutrition and Translational Research In Metabolism, Maastricht University Medical Center, Maastricht, The Netherlands; 5 Department of Biomedical Engineering, Eindhoven University of Technology, Eindhoven, The Netherlands; 6 Subdivision of Rheumatology, Department of Internal Medicine, Maastricht University Medical Center, Maastricht, The Netherlands; 7 Department of Medicine and Life Sciences, Hasselt University, Hasselt, Belgium; 8 Human Performance Laboratory, Faculty of Kinesiology, University of Calgary, Calgary, Canada

**Keywords:** Bone phenotype, Medical imaging, Fracture risk, Osteoporosis, Bone mineral density, Bone microarchitecture

## Abstract

**Purpose of Review:**

Summarize the recent literature that investigates how advanced medical imaging has contributed to our understanding of skeletal phenotypes and fracture risk across the lifespan.

**Recent Findings:**

Characterization of bone phenotypes on the macro-scale using advanced imaging has shown that while wide bones are generally stronger than narrow bones, they may be more susceptible to age-related declines in bone strength. On the micro-scale, HR-pQCT has been used to identify bone microarchitecture phenotypes that improve stratification of fracture risk based on phenotype-specific risk factors. Adolescence is a key phase for bone development, with distinct sex-specific growth patterns and significant within-sex bone property variability. However, longitudinal studies are needed to evaluate how early skeletal growth impacts adult bone phenotypes and fracture risk. Metabolic and rare bone diseases amplify fracture risk, but the interplay between bone phenotypes and disease remains unclear. Although bone phenotyping is a promising approach to improve fracture risk assessment, the clinical availability of advanced imaging is still limited. Consequently, alternative strategies for assessing and managing fracture risk include vertebral fracture assessment from clinically available medical imaging modalities/techniques or from fracture risk assessment tools based on clinical risk factors.

**Summary:**

Bone fragility is not solely determined by its density but by a combination of bone geometry, distribution of bone mass, microarchitecture, and the intrinsic material properties of bone tissue. As such, different individuals can exhibit distinct bone phenotypes, which may predispose them to be more vulnerable or resilient to certain perturbations that influence bone strength.

## Introduction

The mechanisms that lead to bone fragility and subsequent fracture risk are multifaceted. Yet, the current clinical gold standard for diagnosing osteoporosis is centered on one measured bone trait: areal bone mineral density (aBMD) captured with dual-energy X-ray absorptiometry (DXA) [1]. Although aBMD provides insight into bone mass, it does not provide context about the distribution of mass across the bone nor information regarding bone microarchitecture [2]. Consequently, stratification of the population based on aBMD alone fails to identify most individuals who go on to have a fragility fracture [3, 4]. Three-dimensional (3D) medical imaging technologies have made it possible to assess many of the determinants of bone fragility in vivo, improving our understanding of the characteristics that underpin fracture risk beyond aBMD [5, 6]. This includes insight into how whole bone structure and density distribution impact bone strength using computed tomography (CT) [7, 8], alongside an improved understanding of how bone microarchitecture influences bone strength, as measured by high-resolution peripheral quantitative CT (HR-pQCT) [9, 10]. However, even when incorporating this wealth of information about bone structure and volumetric bone mineral density (BMD), the improvement in assessing fracture risk in the general population beyond aBMD has only been incremental [9, 11]. The plateau in improvement may in part be due to the “one-size-fits-all” approach to characterizing bone fragility across the population, rather than recognizing that individuals likely experience different mechanisms of bone loss.

Advanced medical imaging has increased our recognition that there is substantial variability in bone traits across the population. However, there remains the continued practice of treating cohorts as homogeneous when identifying traits that are indicative of bone fragility [12]. Although it is reasonable to presume that common traits exist across individuals who have a fragility fracture, the interindividual variability can overshadow differences reported between fracture and non-fracture cohorts [13]. As such, there is not necessarily a single combination of optimal skeletal traits, but instead, there may be different combinations of these traits, termed bone phenotypes, that can achieve similar functional needs. Recent studies leveraging medical imaging have begun to identify prominent bone phenotypes and stratify individuals based on phenotype to determine whether this approach can help elucidate the different mechanisms that lead to bone fragility [14, 15

In this review, we summarize the latest literature implementing advanced medical imaging to investigate bone phenotypes across the lifespan. We highlight the potential implications a phenotypic approach has for improving fracture risk assessment across the lifespan, particularly focusing on aging and growth, the critical transitionary life stages for skeletal health. Furthermore, we explore the potential benefits of taking a phenotypic approach when assessing metabolic and rare bone diseases to better understand the disease-phenotype interaction. Finally, we provide clinical context into the assessment of skeletal phenotypes, highlighting current imaging strategies that are available in a clinical setting to assess fracture risk using advanced imaging.

## Bone Phenotypes and Their Role in Fracture Risk Assessment

Bone is a complex adaptive system where multi-scale traits (material properties, microarchitecture, geometry) collectively determine a bone’s mechanical properties and resistance to fracture. These traits adapt in a coordinated manner to meet the daily functional needs of an individual, within genetically and physiologically viable constraints [13, 16]. For instance, if one trait is inhibited (e.g., external bone size), it is possible for other traits to adapt (e.g., cortical thickness), to ensure sufficient mechanical function is maintained. Consequently, there is natural variability across the population, where different bone phenotypes meet functional needs. However, when bone homeostasis is disrupted or becomes imbalanced, bone traits may not be able to compensate for each other’s deficits, leading to compromised bone strength and increased fracture risk. Thus, to appropriately determine an individual’s fracture risk, it is insufficient to evaluate bone traits independently; instead, they should be considered in the context of the whole mechanical system [12]. The conceptual framework of bone phenotypes seeks to explore which combinations of bone traits arise more often and to determine how resistant these phenotypes are to short- and long-term fracture risk due to age-related bone loss or to perturbations in bone homeostasis that can be caused by disease or pharmacological therapies. In this section, we discuss current findings regarding bone phenotypes on the macro- and micro-scale that have been identified using advanced imaging and their implications for fracture risk.

### Narrow Versus Wide Bone Phenotypes

Whole bone shape and mineral organization play a crucial role in bone strength and consequently in fracture risk. Characteristics that are typically assessed include cross-sectional area, cortical thickness, and density, either as areal or BMD [17]. When describing the attributes of bone structure and density that are considered favorable for overall bone strength, a generalized characterization is conventionally provided, whereby larger, thicker, and denser bones are seen as advantageous [2, 18, 19]. This is because bone strength in bending is directly proportional to the distribution of mass about the neutral bending axis, while compressive strength also increases with a larger cross-sectional area [20]. Thus, even small increases in the external diameter of a long bone (increased bone size) will improve a bone’s ability to resist loading and meet functional needs [21, 22]. Although characterizing a larger cross-sectional area as a preferred trait is a reasonable approach when considering immediate or short-term bone strength, it does not necessarily take into consideration how the bone will adapt over time, particularly during aging [18, 23].

Several recent studies have demonstrated how external bone size influences the temporal changes in bone strength throughout adulthood. A 14-year longitudinal DXA-based study found that females with narrow versus wide femoral necks had different rates of change in bone mineral content and area during the menopause transition [24]. Specifically, females with narrow femoral necks had smaller losses in bone mineral content (BMC) and greater increases in bone area relative to females with wide femoral necks who faced notable BMC losses without compensatory bone area growth. Furthermore, aBMD before menopause did not predict changes in structure or mass [24]. Another study stratifying females based on wide versus narrow femoral necks identified that there are likely differentiating risk factors for wide versus narrow bone phenotypes [25•]. In females with narrow bones, 80% of the variation in strength was explained by age, weight, and aBMD, while these same risk factors only accounted for half of the variation in strength in females with wide bones [25•]. These findings suggest that the variability in age- or menopause-related trajectories in bone traits can in part be explained by structural bone phenotypes, in this case, external bone size.

**Fig. 1:**
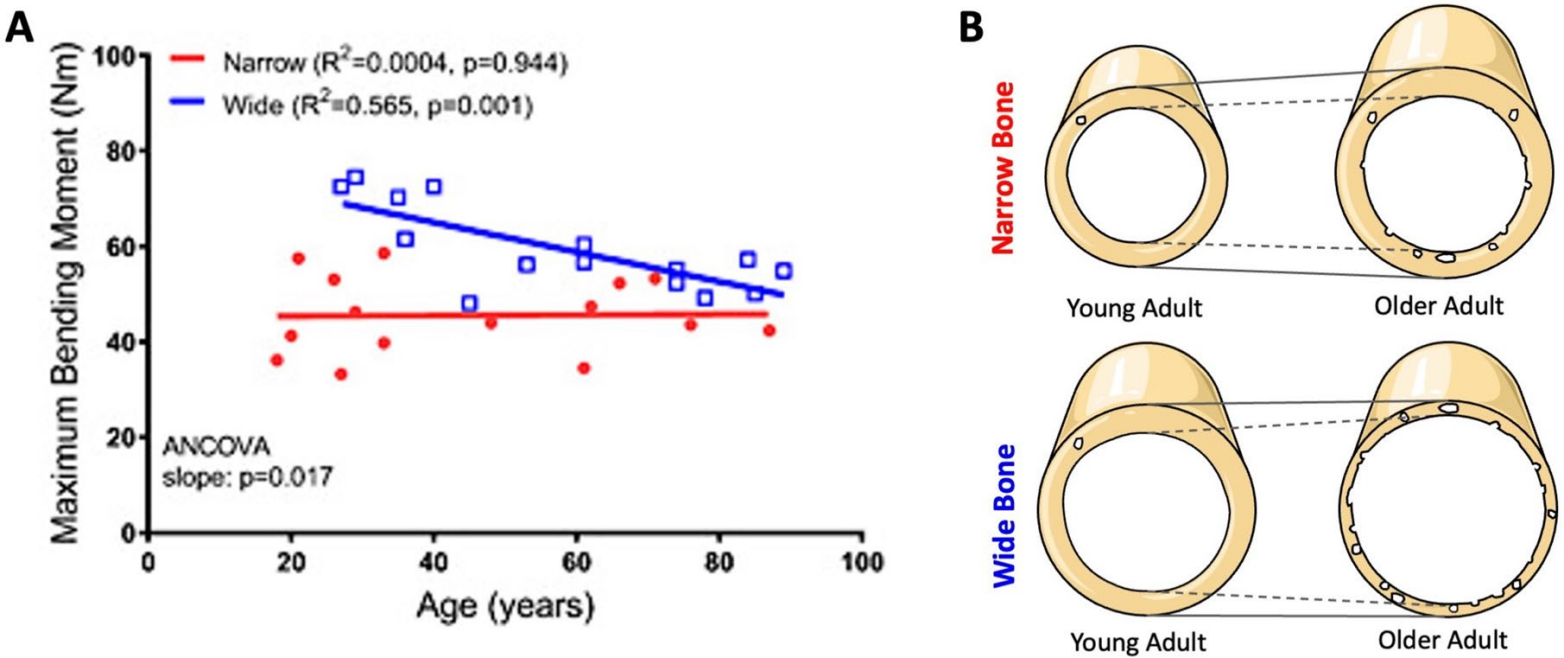
**A** Association between age and bone strength (represented as bending moment) for male radii when grouped according to height-adjusted bone width [26]. **B** Schematic illustrating the structural changes due to aging in wide versus narrow bone phenotypes (adapted from [26])

Similar findings have been found in males between the ages of 18 and 89 years, where cadaveric radii were assessed using peripheral quantitative CT (pQCT) and mechanical testing [26]. When the samples were divided into narrow versus wide bones, young adult males (< 40 years) with wide bones were 54% stronger compared to young adult males with narrow bones, but strength did not differ between older adult males with wide versus narrow phenotypes. In males with wide radii, there was a significant negative correlation between strength and age, while there was no such relationship between strength and age for narrow radii (Fig. 1A) [26]. Comparable findings were identified at the femur, where males with wide bones had age-related declines in whole bone strength, while those with narrow femora had no significant age-related decline in strength [15••]. In both the radius and the femur, this divergent aging process between wide and narrow bones was in part explained by wide bones experiencing a more damaging effect of increasing cortical porosity, due to pore size and spatial location, alongside hindered periosteal expansion. In contrast, narrow bones maintained steady periosteal expansion throughout aging alongside a reduced impact of cortical porosity on bone strength [15, 26].

Combined, these studies highlight evidence that phenotypic subsets of bone structure exist within sexes, particularly that narrow versus wide bones do not share the same age-related trajectories in overall bone strength. When extending the assessment across sexes, phenotypic variation becomes more pronounced, where female bones are not simply a proportionally smaller version of male bones. Rather, females typically have less bone mass and strength than males, after adjustment for body size, and this is driven primarily by a smaller relative cortical area [27, 28]. These insights have implications for assessing short- and long-term fracture risk, as in combination with sexual dysmorphism of the skeleton, wider bones are mechanically stronger at younger ages but experience greater age-related declines in strength when compared to narrow bones (Fig. 1B). Although further investigation is needed to understand the mechanisms driving phenotypic differences in structure and subsequent strength-decline trajectories, accounting for bone size could benefit the assessment of short- and long-term fragility fracture risk.

### Microarchitectural Bone Phenotypes

The advent of HR-pQCT has enabled extensive investigation into the role of bone microarchitecture on fracture risk [29, 30]. Microarchitectural deterioration that underpins increased fracture risk is conventionally characterized by declining BMD, cortical thinning through endocortical resorption, increasing cortical porosity, and deteriorating trabecular microarchitecture [9, 10]. Although these attributes have been consistently linked with heightened fragility fracture risk [11, 31, 32], the approach often implies that these mechanisms of bone loss occur homogeneously across individuals. However, a recent population-based study using HR-pQCT demonstrated the lack of association between cortical bone deterioration (i.e., cortical thinning and increased porosity) and trabecular bone deterioration, in the form of bone void spaces, across the adult lifespan [33]. In fact, several cohort studies have demonstrated using cluster analysis that an elevated fracture risk can arise through deterioration of either cortical or trabecular compartments independently, in both males and females, highlighting that there is not a single microarchitectural phenotype of fragility [34–36].

**Fig. 2:**
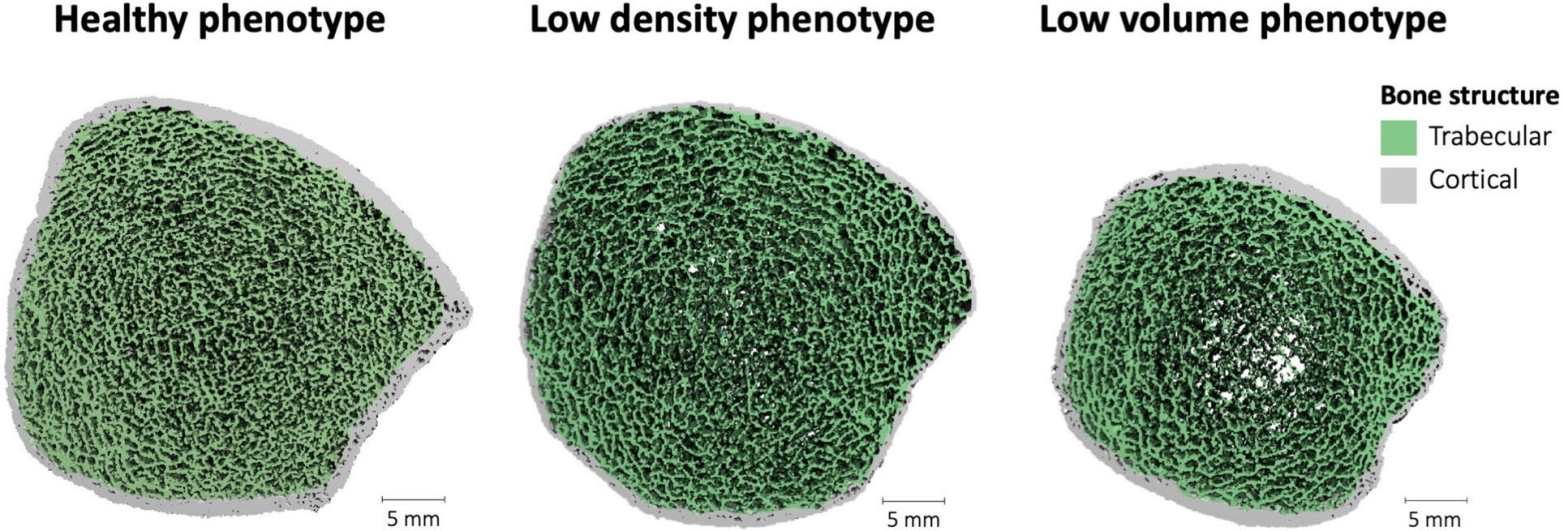
Three-dimensional reconstruction of HR-pQCT scans at the distal tibia depicting bone microarchitecture in the healthy, low density, and low volume phenotypes identified in the Bone Microarchitecture International Consortium (BoMIC) cohort (adapted from [14••])

Building on these insights, the Bone Microarchitecture International Consortium (BoMIC) leveraged fuzzy clustering, a machine learning technique, to determine whether distinct phenotypes of bone microarchitecture could be identified in a large international HR-pQCT cohort of male and female adults (*n* = 6836) [14••]. Three prominent phenotypes were identified in the older adult population, described as *low-density*, *low-volume*, and *healthy* bone phenotypes, based on their defining characteristics in terms of BMD and microarchitecture (Fig. 2) [14••]. Prospective fracture information indicated that each phenotype had differing risks of fragility fracture, where the *low-density* phenotype had the highest fracture risk overall, and that within each phenotype a unique set of bone imaging biomarkers predicted fracture risk [14••]. This study suggests that certain mechanisms of microarchitectural deterioration (e.g., cortical versus trabecular deterioration) may be more detrimental for a specific phenotype, as they may not be able to compensate mechanically through adaptation of other traits. A retrospective study of hip fracture patients further verified a strong relationship between fractures at the hip, a major osteoporotic fracture site, and the *low-density* phenotype, but highlighted sex-specific differences in terms of distribution between male and female hip fracture patients across the microarchitectural phenotypes [37•].

Investigation into microarchitectural bone phenotypes aligned with macro-scale observations of wide versus narrow bone phenotypes, particularly the premise that external bone size plays a notable role in the progression of age-related bone fragility [15, 24, 26]. Specifically, the *low-density* phenotype, which has a larger total cross-sectional area and thinned cortex, had a higher fracture risk compared to the *low-volume* phenotype, which has a smaller cross-sectional area and a thicker cortex. However, even within similar bone sizes, microarchitectural variations exist, as seen between the *low-density* and *healthy bone* phenotypes which have comparable bone size in terms of total cross-sectional area, but exhibit different microarchitectural arrangement [14••]. Given bone deterioration can manifest independently through different mechanisms (e.g., cortical versus trabecular bone loss), fracture risk likely depends on the ability of a bone phenotype to compensate for that form of structural deterioration [38]. However, current phenotypic characterization of bone microarchitecture has been limited to cross-sectional data, and thus longitudinal changes in relation to an individual’s phenotype have yet to be explored. In this sense, it is unknown whether certain microarchitectural phenotypes are more likely to experience a specific form of bone loss, and to what extent the phenotypes identified thus far manifest through aging or disease-related processes.

## Implications of Growth on Bone Phenotypes

Adolescence is a critical period for skeletal development, as 30–50% of peak bone mass, the maximum amount of bone attained in an individual’s life, is accrued during this time [39, 40]. Alongside accumulation of sheer bone mass, adolescent growth is a period when bone shape and microarchitectural characteristics are established. In fact, as much as 60% of the risk of developing osteoporosis later in life can be explained by the peak bone mass an individual attains after adolescent growth has ceased [41–43]. Consequently, adolescence is a critical transitionary stage for bone growth and lifelong fracture risk. In this section, we highlight the insights gained from advanced imaging into the potential impact bone growth has on bone phenotypes identified later in life and consider the short-term consequences of bone growth on fracture risk during adolescence.

### Emergence of Lifelong Bone Phenotypes During Growth

**Fig. 3:**
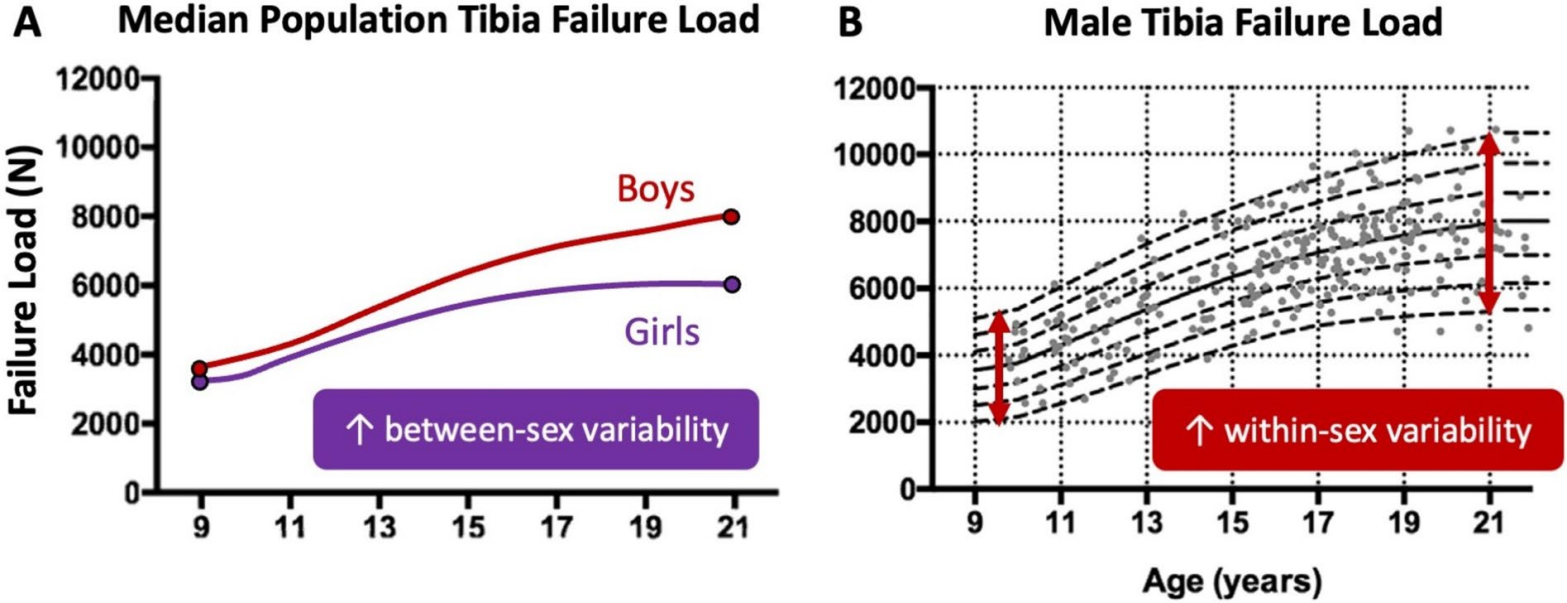
**A** Deviation in median failure load at the distal tibia measured by HR-pQCT between White males and females during growth. **B** Percentile curves of distal tibia failure load in White males during the same time frame, with the increase in variation of bone strength highlighted. Graphs adapted from [45, 48]

Recent studies implementing HR-pQCT and pQCT to study skeletal development across adolescence have clearly established distinct sex-specific trajectories in bone size, microarchitecture, density, and strength [44, 45]. Many inter-sex phenotypic variations seen in adulthood, such as a proportionally lower cortical area in females [27], originate during adolescent growth. For instance, at 10 years of age, the median difference in failure load at the tibia between White males and females is only 9%; however, after growth has slowed (age 21 years), males have a 31% advantage over females (Fig. 3A) [45]. This difference arises because males experience prolonged childhood growth and a greater magnitude of pubertal growth when compared with females [43]. The temporal difference in onset and rate of pubertal growth usually results in greater gains for males in terms of periosteal and longitudinal bone formation, resulting in a phenotype with larger cross-sectional bone area and greater estimated bone strength at diaphyseal sites [46, 47].

Beyond established sex-specific growth trajectories, recent adolescent centile curves developed from a large cohort of adolescent males and females (*n* = 1071) show a pronounced increase in within-sex variability of bone properties on the macro- and micro-scale from the time before the pubertal growth spurt to young adulthood [45]. This surge in variability across the population is in addition to the rapid gains in bone accrual due to growth. For instance, between the ages of 10 and 21 years, the variability (interquartile range) of cortical thickness at the distal tibia increases by 66% in males and 60% in females [45]. Concurrently, the variability in trabecular number increases by 48% in males and 42% females, ultimately resulting in a large variability in bone strength by early adulthood (Fig. 3B) [45]. In contrast, within-sex variability in height does not change to the same extent; between the ages of 10 and 20 years, there is a 9% increase in interquartile range in males and a 4% decrease in variability in females [49]. This discrepancy in the development of skeletal traits (e.g., cortical thickness, trabecular number, height) highlights how structural reorganization during bone growth occurs through different mechanisms, likely establishing the basis of bone phenotypes that are observed later in life.

Despite advances in high-resolution imaging over the past decades, few longitudinal studies have examined how bone microarchitecture, density, and strength adapt during childhood and adolescence, let alone applied a phenotypic approach. Long-term prospective studies are needed to clarify how early skeletal development relates to phenotypes that have been observed later in life, and to what extent intrinsic (e.g., genetics, timing of pubertal onset) and extrinsic (e.g., physical activity, nutrition) factors influence these phenotypic trajectories.

### Dynamic Bone Phenotypes During Growth

During periods of rapid bone growth, more young bone matrix with lower mineralization is present compared with older, denser bone matrix [50]. Adolescence is marked by a transient decrease in cortical BMD during mid-puberty, followed by significant increases, cumulating in an overall rise in BMD of approximately 35–55% [44, 51, 52]. The transient decreases in cortical BMD mid-puberty are underpinned by decreases in cortical thickness and increases in cortical porosity, that then reverse during later adolescence [44, 51]. The trabecular bone compartment also experiences enhancements as a result of growth, in order to more efficiently transfer compressive loads, thereby increasing the mechanical competence of bone [53]. Mixed-longitudinal studies employing HR-pQCT have shown that there is consistently high variability across the adolescent population in trabecular microarchitecture properties (number, thickness, separation) during growth, but the coordinated developmental adaptation of trabecular microarchitecture, alongside cortical traits, leads to a distinct increase in overall load-to-strength ratio [44]. However, similar to cortical bone structure, there are sex-specific differences in microarchitectural changes, especially at the radius [44, 54].

During the pubertal growth spurt, rapid growth can outpace the consolidation of cortical and trabecular bone, resulting in a window of time where the bone is mechanically compromised, temporarily elevating fracture risk [55]. However, prospective studies have struggled to consistently link microarchitecture deficits in adolescents to forearm fractures [56, 57]. It is possible that fracture risk could be influenced by the bone phenotype an individual establishes during this dynamic period of bone growth, particularly if the emerging phenotype further hinders bone’s ability to compensate for the temporary mechanical instability experienced during the pubertal growth spurt. For instance, a cross-sectional study using HR-pQCT showed that females and males (*n* = 115) between the ages 8 and 15 years with a recent low-energy forearm fracture had thinner cortices and lower cortical area at peripheral sites than those without a fracture [56, 58]. Prospective studies further suggest that females who fracture their forearms have significantly lower trabecular BMD that persists for at least several years following fracture [56, 59]. In contrast, prospective and follow-up assessment of cortical and trabecular properties are not consistent in adolescent males [56, 57], suggesting sex differences in prevalent phenotypes that are at risk of fracture during bone growth [56, 57].

Overall, there is broad consensus that bone growth during adolescence is important for establishing long-term skeletal health, and recent population-based studies have provided valuable insight into bone development on the micro-scale. However, long-term prospective studies are needed to clarify skeletal phenotypes associated with fractures in males and females during adolescent growth.

## Metabolic and Rare Bone Diseases in the Context of Bone Phenotypes

Assessment of metabolic and rare bone diseases using advanced medical imaging has been of increasing interest, as these diseases alter bone characteristics through specific mechanisms, leading to increased fracture risk. In theory, gauging fracture risk within a specific disease should be more precise than in the broader population, given it is driven by a specific pathology. Yet, in many cases, bone traits among individuals within a disease are as highly variable as in healthy cohorts, confounding our ability to assess who is at risk of fracture and limiting the ability to develop tailored interventions [60–62]. The heterogeneity in bone traits within a disease may in part be attributed to the interplay between the disease and an individual’s bone phenotype, termed the “disease-phenotype” interaction. In this context, an individual may be more or less resilient to the degenerative effects of a disease, depending on the bone phenotype prior to disease onset. Should this be the case for certain diseases, an understanding of disease-phenotype interactions could help elucidate who will be the most adversely impacted as a disease progresses, enabling interventions to be targeted towards these individuals. Although conceptually compelling, the interplay between bone phenotypes and bone diseases has not yet been explored, and it does not necessarily apply to all diseases. The following section will discuss a few metabolic and rare bone diseases that have been studied extensively in recent years with advanced medical imaging, summarize the insights gained, and highlight how a phenotypic approach may enhance the understanding of fracture risk in these populations.

### Metabolic Bone Diseases

**Fig. 4:**
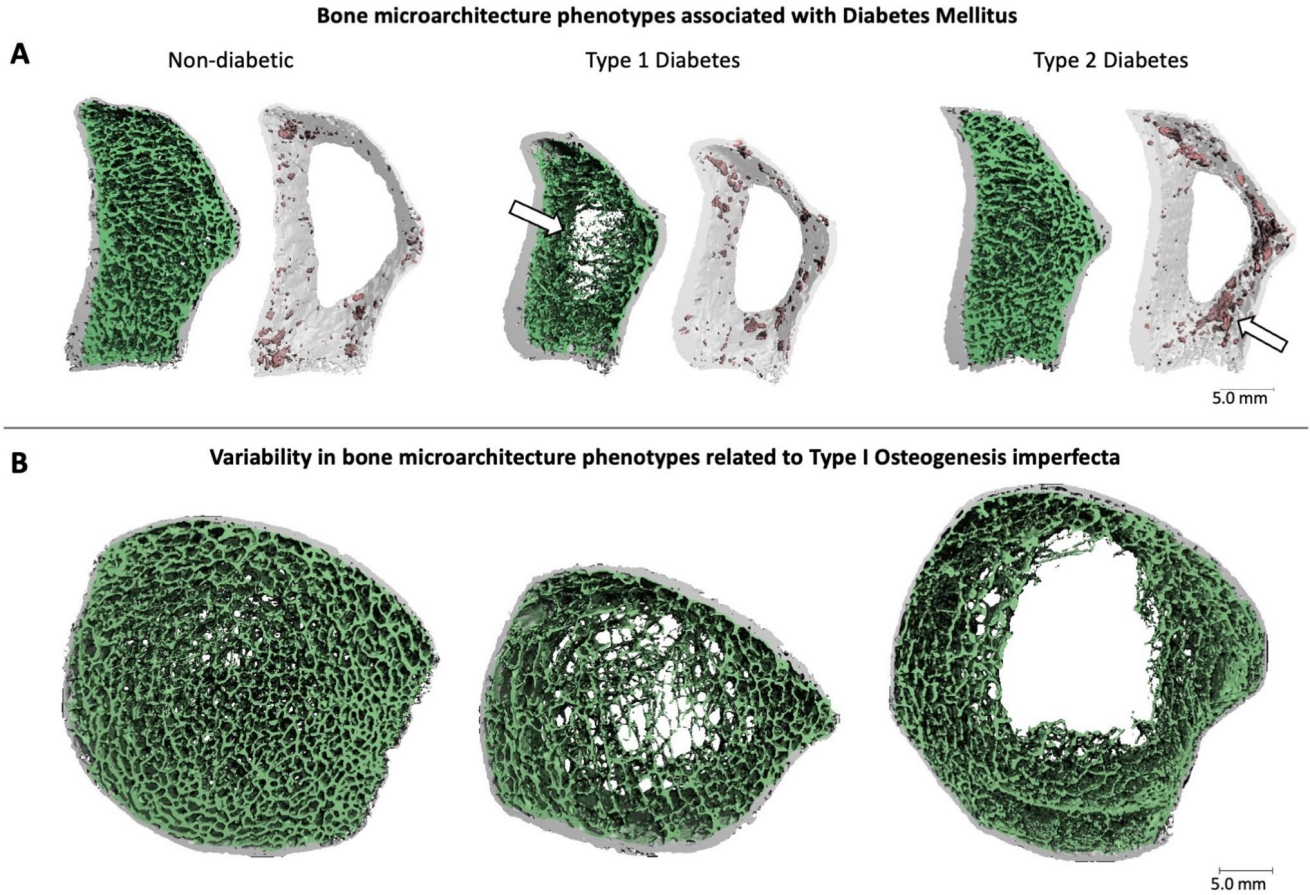
**A** Three-dimensional HR-pQCT reconstructions of distal radius scans showing examples of bone phenotypes identified through the meta-analysis [65•] in older adult females, aged 79–80, without diabetes, type 1 diabetes (trabecular deterioration), and type 2 diabetes (cortical porosity). The left figure of each example depicts trabecular (green) and cortical (grey) microarchitecture and the right figures of each example depict porosity (red) in the cortical compartment (transparent grey). Examples are from population-based cohorts courtesy of the Bone Imaging Lab, Calgary, Canada [37, 48]. **B** Three-dimensional HR-pQCT reconstructions of distal tibia scans showing examples of the variability of bone phenotypes in females with type I osteogenesis imperfecta [67]

Diabetes mellitus, categorized primarily as type 1 (T1D) and type 2 (T2D) based on pathophysiology, is among the most common diseases affecting bone metabolism. Both T1D and T2D are associated with increased fracture risk due to a combination of different cellular and molecular mechanisms that can lead to alterations at the cellular, matrix, and structural level [63]. Given the complexity of these mechanisms acting on bone, it is not surprising that aBMD alone does not fully explain the increased fracture risk in patients with diabetes [64]. A recent meta-analysis of HR-pQCT studies found that T1D (4 studies) was associated with significant trabecular deterioration at the distal radius, evident by lower trabecular BMD and number and increased inhomogeneity [65•]. In contrast, T2D (12 studies) was linked to intra-cortical deterioration at the radius, identified by a higher cortical porosity (Fig. 4A) [65•]. Interestingly, structural degeneration was not detected at the tibia, suggesting that mechanical loading may counteract diabetes-induced bone deterioration. Despite these insights, establishing a distinct bone phenotype for T1D and T2D remains challenging [60]. The meta-analysis highlighted variability in findings across populations, further emphasized by a recent study that found contradictory results in a large cohort of participants with T1D [66]. Reasons for the lack of consensus likely stem from the broad range of pre-existing bone phenotypes among individuals who develop diabetes, underscored by the heterogeneity in age, ethnicity, and sex of studied populations [29, 63]. Thus, the impact of diabetes on an individual’s bone strength, and consequently fracture risk, may depend on their pre-existing bone phenotype and its adaptive ability to compensate mechanically for disease-driven bone degeneration. For example, as mentioned above, wider bones exhibit greater strength-decline trajectories during aging due to increasing cortical porosity and hindered periosteal expansion than narrow bones [26]. Thus, an individual with a wide bone phenotype may potentially be more severely afflicted by the additive effects of T2D on pore expansion than an individual with a narrow bone phenotype, leading to an increase in fracture risk in a wide bone phenotype. Conversely, a narrow bone that maintains periosteal expansion throughout aging may be more capable of compensating structurally for T2D-induced cortical porosity. However, future research, particularly longitudinal studies, are necessary to understand the potential for disease-phenotype interactions and implications on diabetic fracture risk.

Chronic kidney disease (CKD) and glucocorticoid-induced osteoporosis (GIOP) are both metabolic bone diseases that can cause severe bone deterioration, albeit through different mechanisms. CKD is a multifaceted condition, of which increased fracture risk constitutes just one part of the systemic effects of the disease [68, 69]. Deterioration of bone microarchitecture measured by HR-pQCT is already observed in the early stages of CKD [70], with more pronounced alterations in patients at more advanced stages [71]. Nevertheless, the specific nature of CKD-related bone loss (trabecular versus cortical) varies across studies using QCT and HR-pQCT [72•], and consequently, the sensitivity of DXA-based aBMD in assessing fracture risk is limited [61]. In contrast, GIOP is a direct consequence of glucocorticoid treatment, often prescribed for conditions such as rheumatological conditions [73] or lung diseases [74]. Reduced BMD, microarchitecture, and strength have been reported in postmenopausal females on glucocorticoid treatment when compared to healthy controls [73, 74]. Several studies have shown that BMD, bone microarchitecture, and strength from QCT and HR-pQCT can discriminate vertebral fractures in glucocorticoid-treated adults [75, 76]. While GIOP severity often correlates with treatment dose and duration [77], still some patients on a low glucocorticoid dose can have a higher rate of bone loss than patients on a higher dose [78]. Although the cellular and molecular pathophysiology of GIOP have been extensively studied, the variation in bone response to glucocorticoid treatment remains poorly understood [78]. For both CKD and GIOP, the mechanisms of bone loss might differentially impact the mechanical integrity of the bone phenotypes discussed throughout this review. Enhancing our grasp on how disease-driven bone degeneration affects specific phenotypes may help pinpoint those at the highest risk of rapid strength decline, guiding targeted interventions for both conditions.

### Rare Bone Diseases

Osteogenesis imperfecta (OI) is a rare bone condition with unique bone phenotypes stemming from a genetic collagen deficiency. The skeletal phenotypes among individuals with OI are heterogenous, reflective of the genetic variability of the condition (Fig. 4B). Several studies have used pQCT, and more recently HR-pQCT, to characterize bone phenotypes in OI. Imaging with pQCT has found that bone area and BMD can be low, normal, or high relative to healthy controls, dependent on the skeletal site [79–81]. More recently, HR-pQCT has consistently shown deterioration in the trabecular microarchitecture of patients with OI [67, 82–84]. Generally, bone characteristics vary between OI subtypes and genetic mutations. A recent large cohort study employed HR-pQCT to assess bone microarchitecture and strength relative to age- and gender-matched references and showed considerable interindividual heterogeneity, suggesting that existing OI classifications might not capture the full range of skeletal diversity [67]. It remains unknown whether subgroups based on bone phenotype may exist in OI and to what extent medical imaging can capture phenotypic variability, given the genetic impairment of this condition acts on the material properties of the bone [85].

Other rare conditions with notable interindividual bone phenotype variability include pregnancy- and lactation-induced osteoporosis [86], and inborn errors of metabolism that affect bone such as hypophosphatasia and X-linked hypophosphatasia, Gaucher disease, and Pompe disease [29, 87, 88]. However, advanced imaging data is limited in these populations.

## Clinical Implications of Skeletal Phenotypes

Fractures occur when the load on bone exceeds its capacity to resist fracture and are the result of the presence of bone- and extra-skeletal fracture risk factors. In this context, around 30 years ago, the bone-related phenotype was described in general terms as osteoporosis and defined broadly as “a disease characterized by low bone mass and microarchitectural deterioration of bone tissue, leading to enhanced bone fragility and a consequent increase in fracture risk” [89, 90]. Despite the availability of guidelines on medications and lifestyle for fracture prevention, still many individuals worldwide suffer a fragility fracture [91].

In the context of assessing osteoporotic fracture risk, an individual’s bone phenotype is reduced to a singular trait: aBMD measured by DXA. Areal BMD has a high specificity to predict fractures, meaning that individuals with exceptionally low bone mass (i.e., a *T*-score ≤  − 2.5) are identified to have an osteoporotic phenotype and thus have a high risk of fractures [92, 93]. In contrast, aBMD has a low sensitivity, meaning that most patients who fracture do not have an osteoporotic phenotype according to the aBMD diagnostic threshold. Consequently, when only aBMD is used to phenotype bone and the basis for intervention thresholds, individuals with the osteoporotic phenotype will be treated, regardless of fracture status, while most individuals who will go on to fracture will not receive treatment [92]. This raises the question as to how to improve osteoporosis screening and what other relevant risk factors can be clinically identified.

### The Clinical Implications of Macro- and Microarchitectural Phenotypes

Fracture risk depends on the combined structural and material properties of bone that are not fully captured by aBMD. For example, patients with a fracture have some form of deteriorated microarchitecture (the second part of the WHO definition of osteoporosis), which worsens with age [10]. Prospective studies have shown that combined assessment of cortical and trabecular bone microarchitecture by HR-pQCT improves overall fracture prediction beyond aBMD [9, 94, 95]. Furthermore, the differentiation of patients with severe microstructural deterioration could have important implications for the decision on therapeutical interventions regarding the use of bone-preserving antiresorptive and bone-forming anabolic drugs at any level of aBMD, such as those with wide versus narrow bones. However, the availability of HR-pQCT for clinical application is still limited worldwide, and the clinically available CT systems cannot yet resolve cortical and trabecular microarchitecture to the same level as HR-pQCT [29]. Consequently, alternative strategies need to be employed clinically to assess and manage fracture risk.

### Using Fracture Events to Broaden the Osteoporotic Phenotype

The WHO definition of osteoporosis also includes the presence of a fragility fracture as a sign of osteoporosis. In this context, a history of fragility fracture, regardless of fracture location is an indicator of bone failing to meet its day-to-day functional needs, and thus what can be considered a clinical phenotype of fragility [96–99]. Vertebral fractures (VF) are the most frequent osteoporotic fractures [100•] and considered a hallmark of decreased bone quality [101]. This is perhaps because most VFs arise from daily activities that overload the vertebrae rather than from a fall-induced fracture [102]. As they occur most often (in two-thirds of cases) subclinical, imaging of the spine is the only way to have a full VF history [103]. Vertebral Fractures Assessment (VFA) has been shown to enhance fracture prediction beyond aBMD [104] and beyond FRAX [105], indicating that vulnerability to having a VF is likely a consequence of combined structural and density traits that make a up an individual's bone phenotype. Opportunistic identification of VFs from CT scans performed for other medical reasons is one of the emerging developments for assessing fracture risk and is a promising means to identify definite cases of bone fragility in the population [106]. Conventional computational methods and machine learning-based algorithms are being developed to facilitate (semi)automatic identification of VFs, through a combination of measuring aBMD, volumetric BMD, and biomechanical properties estimated from finite element analysis [107]. These new CT-based algorithms still need to be integrated into the clinical workflow and further validated with respect to patient management [108, 109], but once implemented have potential to broaden identification of individuals with advanced fragility so that the mechanisms leading to VF can be better understood.

### Fracture Risk Phenotypes

Patients with a recent fracture have a wide array of bone- and fall-related risks beyond aBMD that can be used as an indirect means to establish bone fragility phenotypes [110–112]. Risk factors besides fracture history that are associated with short- and long-term fracture risk include various comorbidities, fall history, diseases, and medications [111, 113–118]. Up to 26 risk factors have been included in fracture risk algorithms such as FRAX, Garvan, and QFracture, to stratify the population into what can be considered “fracture risk phenotypes.” However, the intervention threshold for FRAX (when aBMD is included) is defined based on the mean 10-year risk of subjects with a fracture, implying that half of patients with a recent fracture will not meet the intervention threshold based on FRAX phenotyping. FRAX can be adapted further for other risk factors, but the accuracy of multiple adjustments is unclear [119]. Although fracture risk assessment tools provide a readily available approach for stratifying the population to assess fracture risk, they cannot provide interpretation into the mechanisms leading to bone fragility. Consequently, the approach is limited to phenotyping fracture risk, and not phenotyping bone in a manner that could offer insight into targeted strategies for treatment.

## Conclusion

The idea that different combinations of structural traits can lead to bone fragility is not a new notion, but recent advancements in medical imaging have rejuvenated interest and enhanced our ability to explore in greater detail the differing mechanisms leading to fracture [25, 120–122]. Bone fragility is not solely determined by density but by a combination of bone geometry, distribution of bone mass, microarchitecture, and the intrinsic material properties of bone tissue. Each of these traits can be influenced by a myriad of genetic, hormonal, nutritional, and mechanical stimuli throughout an individual’s life. As such, different individuals may exhibit distinct bone phenotypes that predispose them to be more vulnerable or resilient to certain perturbations that influence bone strength. In this sense, skeletal phenotypes are not necessarily groups of traits that define “healthy” versus “fragile” bone but rather seek to identify the combinations of bone properties that arise more frequently together. With this approach, a more targeted strategy can be taken to understand how each individual would respond to perturbations that may disrupt the skeletal system’s ability to maintain a system-level homeostasis [13].

Overall, understanding these bone phenotypes is crucial, not just for predicting fracture risk, but also for effectively tailoring therapeutic and lifestyle interventions to individuals. While aBMD remains a cornerstone for osteoporosis diagnosis, it is clear that a more comprehensive understanding of bone health requires a deeper assessment of bone phenotypes that arise across the population and throughout the lifespan. Advanced medical imaging, combined with emerging data-driven computational techniques, offers a promising path forward in this endeavor, with the potential to transform our approach to bone health and fracture prevention.

## Data Availability

Not applicable.
